# Immunotherapy in genitourinary malignancies

**DOI:** 10.1186/s13045-017-0457-4

**Published:** 2017-04-24

**Authors:** Kathan Mehta, Keyur Patel, Rahul A. Parikh

**Affiliations:** 1 Department of Medicine, University of Pittsburgh Medical Center, Pittsburgh, PA USA; 2 University of Pittsburgh Cancer Institute, Pittsburgh, PA USA; 3 Division of Hematology/Oncology, Department of Medicine, UPMC Cancer Pavilion, 5th Floor, 5150 Centre Avenue, Pittsburgh, PA 15232 USA

**Keywords:** Immunotherapy, Genitourinary malignancy, Checkpoint inhibitor, PD-1, PD-L1, CTLA-4, Sipuleucel-T, Interleukin, Cancer, Kidney, Prostate, Bladder, Nivolumab, Pembrolizumab, Atezolizumab, Durvalumab, Ipilimumab, BCG, PROSTVAC

## Abstract

Treatment of cancer patients involves a multidisciplinary approach including surgery, radiotherapy, and chemotherapy. Traditionally, patients with metastatic disease are treated with combination chemotherapies or targeted agents. These cytotoxic agents have good response rates and achieve palliation; however, complete responses are rarely seen. The field of cancer immunology has made rapid advances in the past 20 years. Recently, a number of agents and vaccines, which modulate the immune system to allow it to detect and target cancer cells, are being developed. The benefit of these agents is twofold, it enhances the ability the body’s own immune system to fight cancer, thus has a lower incidence of side effects compared to conventional cytotoxic chemotherapy. Secondly, a small but substantial number of patients with metastatic disease are cured by immunotherapy or achieve durable responses lasting for a number of years. In this article, we review the FDA-approved immunotherapy agents in the field of genitourinary malignancies. We also summarize new immunotherapy agents being evaluated in clinical studies either as single agents or as a combination.

## Background

**Fig. 1:**
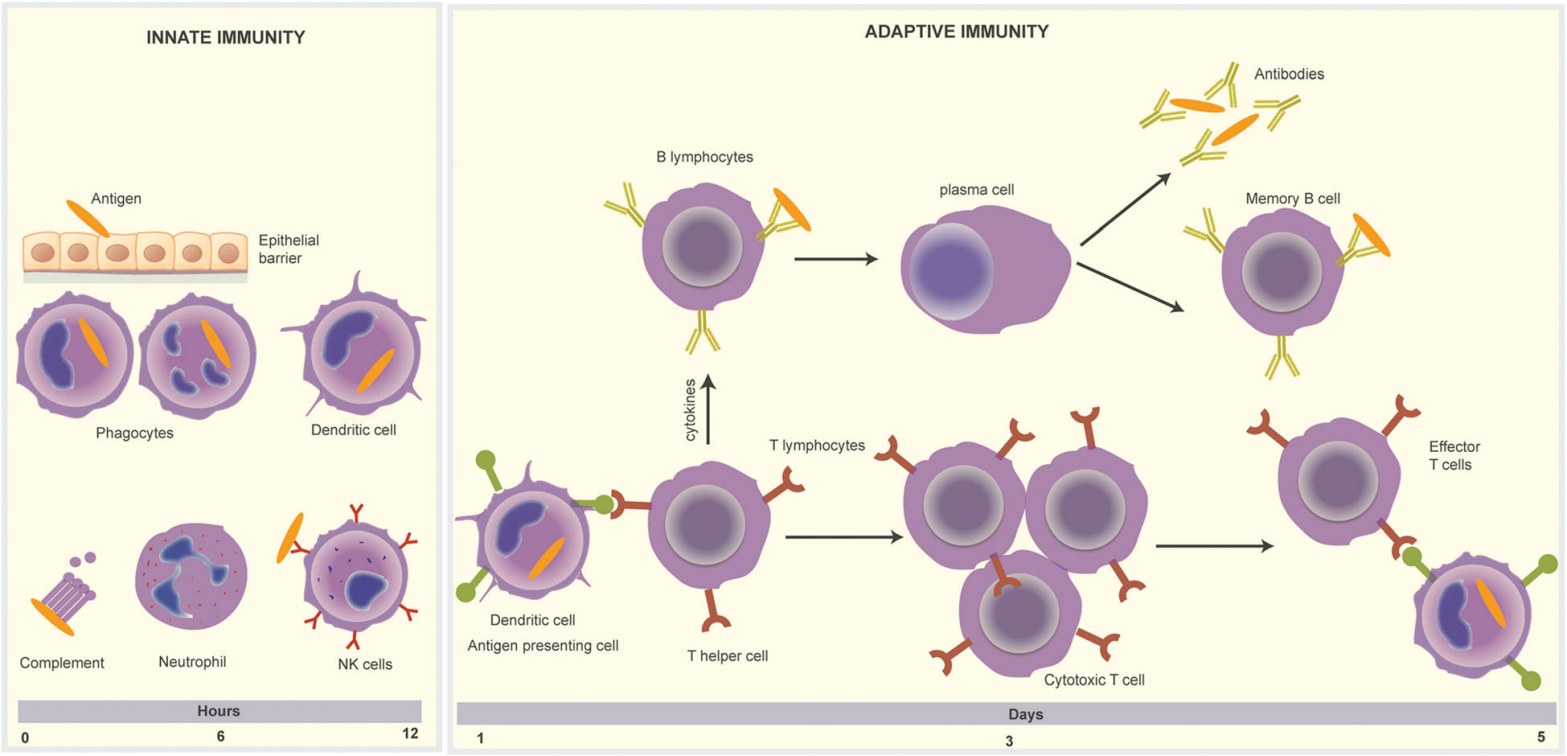
The basic components of the innate and adaptive immune responses to infection and cancer. Innate immune response includes dendritic cells, which are involved in antigen presentation, neutrophils and phagocytes, and activation of the complement system. The adaptive immune response leads to activation of B lymphocytes, which produce specific antibodies and T lymphocytes involved in cytokine release, direct cytotoxicity and retention of memory for the antigens

**Fig. 2:**
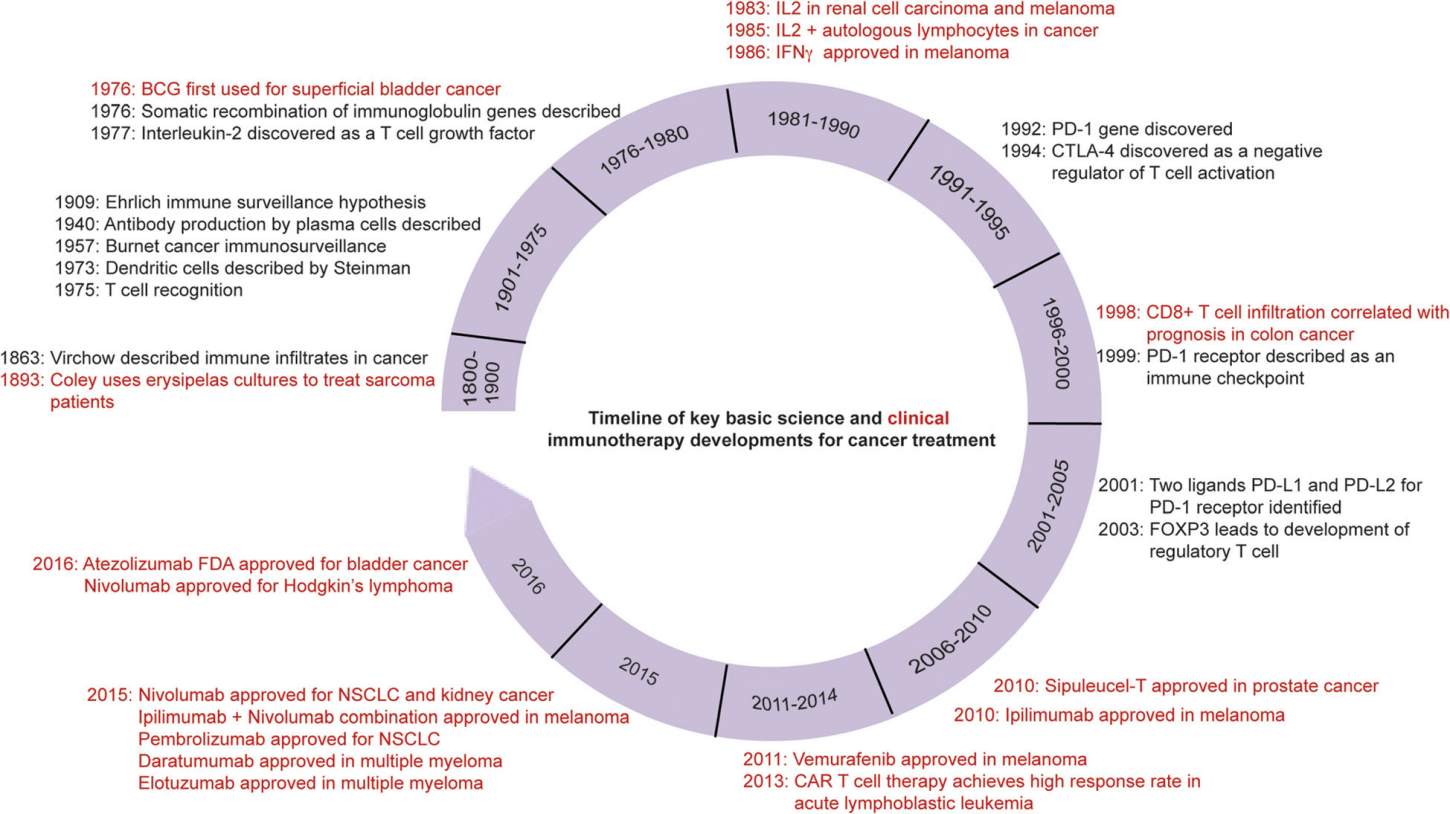
A timeline of important clinical and translational events and timelines in the evolution of cancer immunotherapy. *Black* represents basic science discoveries and *red* represents clinical or translational discoveries

**Fig. 3:**
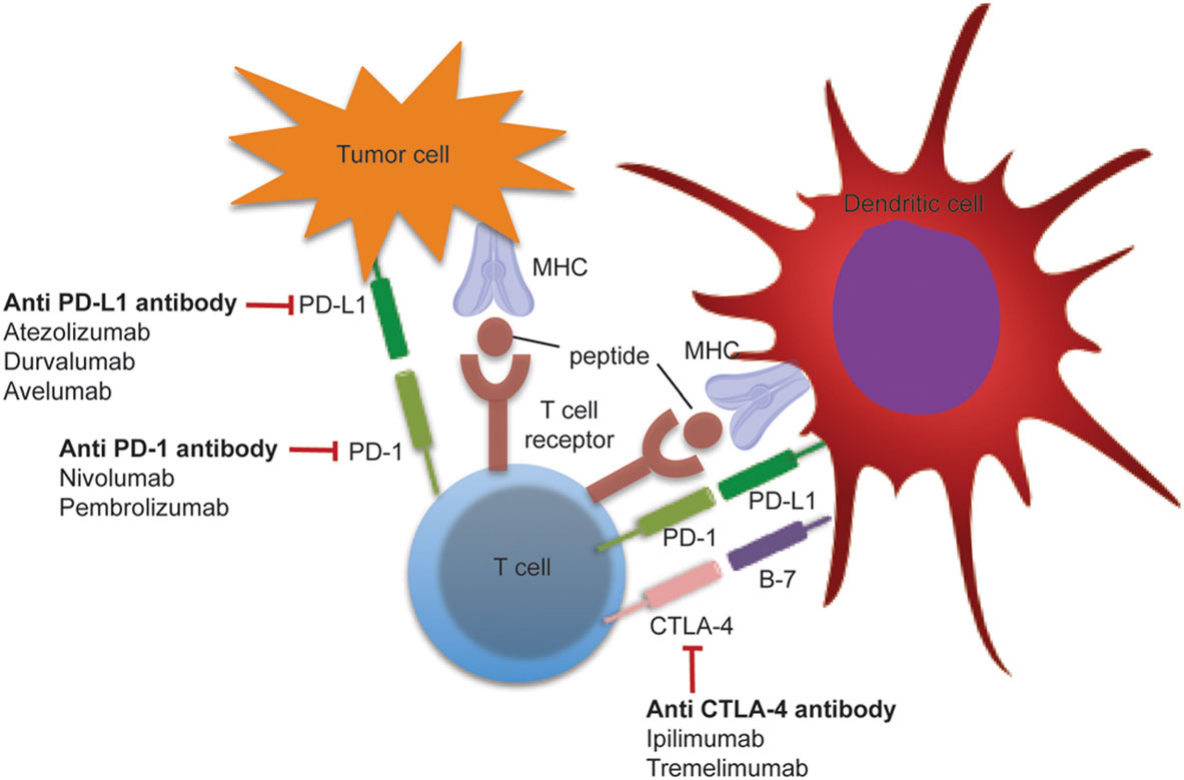
Immunotherapies and their sites of action

The immune system is the body’s main defense mechanism against cancer and infections and consists of innate and adaptive immunity. The innate and adaptive immune responses play a major role in cancer prevention and also retarding cancer progression. The basic components of innate and adaptive immune response are depicted in Fig. 1. The immunological memory against the cancer antigens can lead to long-lasting remission and halt the cancer progression. More than a century back, Virchow studied the role of the immune system, inflammation, and response to cancer [1]. Cancer cells can evade detection and eradication by the immune system by reducing antigen expression, secreting immune-suppressive cytokines, or upregulating inbuilt inhibitory signals. Cancer immunotherapy encompasses a broad variety of agents, which can stimulate, enhance, and modulate the immune system to detect and destroy cancer cells. Immunotherapy agents fall under two categories: non-specific and specific or directed agents. Non-specific therapy includes interferon alpha (IFN-α), various interleukins, cytokines, and vaccines. In contrast, specific immunotherapy includes immune checkpoint inhibitors, which target immune checkpoints (programmed death 1 (PD-1), programmed death ligand 1 (PD-L1), cytotoxic T-lymphocyte-associated antigen 4 (CTLA-4), lymphocyte function-associated antigen 3 (LFA-3)). This review article provides information on US Food and Drug Administration (FDA)-approved immunotherapies used in the treatment of genitourinary cancers. We also summarize ongoing immunotherapy studies that hold promise in effective treatment of genitourinary cancers. Figure 2 reviews important clinical and translational events and timelines in the evolution of cancer immunotherapy (adapted from Lesterhuis et al. [2]). Recently approved checkpoint inhibitors are depicted in Fig. 3.

## Literature review

### Renal cell carcinoma

In the USA, approximately 62,700 new cases of renal cell carcinoma (RCC) will be detected with an estimated 14,240 deaths in the year 2016 [3]. About two thirds of patients who present with localized tumor (stages I–III) can be cured by surgical intervention (radical nephrectomy). These patients have an excellent prognosis with a 5-year survival rate of 80–90% [3]. Patient with distant metastases has a poor prognosis with a 5-year survival rate of 10–12% [3]. A number of immunotherapy agents have been approved by the FDA for the treatment of metastatic renal cell carcinoma (mRCC) and are reviewed below.

#### FDA approved agents: first-line therapy

##### High-dose interleukin 2

The US FDA approved high-dose interleukin (HD-IL2) in 1992 for the treatment of mRCC on the basis of seven phase II clinical trials [4–10]. Interleukin-2 is a naturally occurring cytokine with the ability to expand and differentiate T cell populations with antitumor activity.

In phase II clinical trial, a total of 255 patients with mRCC were treated with HD-IL2 (600,000 or 720,000 IU/kg) every 8 hourly up to 14 consecutive doses for 5 days [4]. A second cycle was repeated after 5–9 days, and courses were repeated every 6–12 weeks in patients with stable disease or partial responses. The overall response rate (ORR) was 14% with complete response (CR) seen in 5% patients and partial response (PR) in 9% of patients [11]. Median duration of PR was 19 months (Table 1). Baseline Eastern Cooperative Oncology Group (ECOG) performance status (PS) was the only prognostic factor for predictive of response to HD-IL-2. Side effects with HD-IL2 are extremely common and may be severe; thus, administration of HD-IL2 is recommended in specialized centers trained to manage its side effects. Common side effects associated with HD-IL-2 were hypotension, fever and chills, anemia, nausea and vomiting, diarrhea, mental status changes, elevated liver enzymes and bilirubin, elevated BUN and creatinine, dyspnea, and pruritus. Most of the severe toxicities were associated with capillary leak syndrome. Grade 3 or 4 AEs associated with HD-Il2 administration include hypotension, oliguria/anuria, nausea/vomiting, and mental status changes. There is evidence to show that centers, which perform high volumes of HD-IL2 administration, have lower inpatient mortality related to its toxicity [12].

In a prospective study, 120 eligible patients were enrolled to evaluate whether the ORR of patients with mRCC with “good” predictive pathologic features based on an “integrated selection” model [ISM (clear-cell histology and carbonic anhydrase-9 (CA-9) IHC staining] was significantly higher than the ORR of a historical, unselected population [13]. The independently assessed ORR was 25% (30/120, 95% CI, 17.5–33.7, *p* = 0.0014; 3 patients achieved complete responses, 27 achieved partial responses) and was higher than the historically observed ORR with 11% patients remaining disease free at 3 years. Median OS was 42.8 months.

##### Interferon with Bevacizumab

Interferon alpha (IFN-α) is a cytokine with immune-modulatory and anti-proliferative activity in mRCC. Bevacizumab is a monoclonal, recombinant, humanized, anti-VEGF antibody (vascular endothelial growth factor) and has activity against mRCC. IFN-α in combination with bevacizumab was approved as first-line therapy by the US FDA for the treatment of patients with metastatic RCC, based on a prospective, randomized, multicenter phase III trial.

A total of 732 patients were randomly assigned in two groups to receive either bevacizumab (10 mg/kg intravenously every 2 weeks) with IFN-α (9 million units SC 3 times/week) or IFN-α monotherapy [14, 15]. The median PFS was 8.5 months for bevacizumab plus IFN (95% CI, 7.5 to 9.7 months) compared to 5.2 months (95% CI, 3.1 to 5.6 months) for IFN-α monotherapy. ORR was higher, 25.5% for the combination compared to 13.1% to IFN-α monotherapy. The median OS (primary end point) was 18.3 months for the combination compared to 17.4 months for IFN-α. PFS and OS were greater in patients who developed grade ≥2 hypertension (PFS 13.2 vs. 8.0 months, OS 41.6 vs. 16.2 months). Bevacizumab with IFN-α was commonly associated with fatigue, anorexia, nausea, proteinuria, neutropenia, and hypertension. More grade 3 or 4 AE including hypertension, anorexia, fatigue and proteinuria occurred with bevacizumab with IFN-α. In this study, OS-favored combination of bevacizumab with IFN-α but did not meet the pre-defined criteria for significance.

In a double-blind, multicenter, phase III trial, a total of 649 patients with untreated mRCC were randomized to two groups of bevacizumab plus IFN-α (*n* = 327) and IFN-α plus placebo (*n* = 322) [16]. Median OS (primary end point) with bevacizumab and IFN-α was 23.3 months, and IFN-α with placebo was 21.3 (stratified hazard ratio (HR) = 0.86; 95% CI, 0.72 to 1.04; *p* = 0.1291). A majority of patients (>55%) in both groups were treated with at least one post-protocol agent, which may confound the OS analysis. At the planned interim analysis, median PFS was significantly longer with bevacizumab with IFN-α vs. IFN-α/placebo (10.2 vs. 5.4 months; HR 0.63; *p* < 0.001 un-stratified) and ORR 31 vs. 13%, respectively (*p* < 0.001 un-stratified).

In another prospective, randomized, multicenter phase III trial, a total of 791 clear-cell mRCC patients were enrolled and randomly assigned in two groups of bevacizumab plus temsirolimus (*n* = 400) or bevacizumab plus IFN-α (*n* = 391) [17]. Median PFS (primary end point) with bevacizumab and temsirolimus combination was 9.1 months compared to 9.3 months in bevacizumab and IFN-α [HR, 1.1; 95% CI, 0.9 to 1.3; *p* = 0.8]. OS (25.8 vs. 25.5 months; HR, 1.0; *p* = 0.6) and ORR (27.0 vs. 27.4%) were not significant in bevacizumab with temsirolimus and bevacizumab with IFN-α respectively. Common adverse effects with bevacizumab and temsirolimus were rash, hypercholesterolemia, mucosal inflammation, stomatitis, hypophosphatemia, and hyperglycemia whereas pyrexia, neutropenia, and myalgia were more common with bevacizumab and IFN-α. This study showed that temsirolimus with bevacizumab combination therapy was not superior to bevacizumab with IFN-α.

#### Second line and beyond

##### Nivolumab

Nivolumab is a programmed death 1 (PD-1) checkpoint inhibitor. In a large phase 1 study, 296 patients with lung, kidney, prostate, or melanoma cancer patients were treated with increasing doses of nivolumab. For the kidney cancer cohort, objective responses were seen in 4 of 17 patients (24%) at 1 mg/kg dose and 5 of 16 (31%) patients at 10 mg/kg dose; stable disease was seen in an additional 9 patients (27%). Five patients had a very durable response lasting for more than 1 year. Thus, nivolumab demonstrated excellent clinical activity in metastatic renal cell carcinoma [18].

Subsequently, nivolumab was compared to everolimus in a randomized, phase III study, in patients with advanced renal cell carcinoma who had been treated previously with anti-angiogenic therapy and/or cytokine therapy (CheckMate 025). A total of 821 patients with renal cell carcinoma were randomized in a 1:1 ratio to receive intravenous nivolumab 3 mg/kg of body weight every 2 weeks or oral everolimus tablet (10 mg) once daily [19]. The median OS (primary endpoint) was 25.0 months with nivolumab compared to 19.6 with everolimus. The HR for death with nivolumab vs. everolimus was 0.73 (98.5% confidence interval (CI), 0.57 to 0.93; *p* = 0.002). The median PFS 4.6 months with nivolumab compared to 4.4 with everolimus. The ORR was statistically superior with nivolumab compared to everolimus (25 vs. 5%; odds ratio: 5.98, *p* < 0.001). Nivolumab use was commonly associated with fatigue, nausea, pruritus, diarrhea, anorexia, and rash. Fewer grade 3 or 4 adverse events occurred with nivolumab compared with everolimus. At the interim analysis, health-related quality of life (HRQoL) was evaluated between nivolumab and everolimus using validated scales. More patients had a clinically meaningful improvement in HRQoL, achieved over a shorter duration with nivolumab compared to everolimus [20]. Interestingly, as noted with sipuleucel-T in prostate cancer, use of nivolumab did not improve PFS, though ORR and OS were statistically better with nivolumab compared to everolimus. Nivolumab also had a much better overall side-effect profile compared to everolimus and has quickly been incorporated into treatment strategies for metastatic renal cell carcinoma. A number of combination therapies with nivolumab including ipilimumab (NCT02231749) or VEGF tyrosine kinase inhibitors (NCT01472081) are currently ongoing.

## Non FDA-approved therapies

### Atezolizumab

Atezolizumab, humanized PD-L1 antibody, was evaluated in patients with metastatic RCC in phase I trial to assess safety, efficacy, and immune correlates. This study enrolled 70 patients with mRCC (63 clear-cell RCC and 7 non-clear-cell RCC), who received intravenous atezolizumab every 3 weeks [21]. Expression of PD-L1 was measured as 0, 1, 2, or 3 based on their staining on tumor cells and tumor-infiltrating immune cells (IC). Patients with clear-cell RCC (*n* = 62) had a median OS of 28.9 months, median PFS of 5.6, and ORR of 15%. ORR as assessed by PD-L1 expression was higher for IC1/2/3 positive tumors 18% compared to IC0 (negative tumors) of 9%. ORR for Fuhrman grade 4 and/or sarcomatoid histology was 22%. Atezolizumab is commonly associated with fatigue, poor appetite, arthralgia, rash, nausea, anemia, chills, diarrhea, pruritus, and pyrexia. Grade 3/4 AEs included fatigue (4%), anemia (4%), and hypophosphatemia (3%). Response to atezolizumab showed decrease in circulating plasma markers and acute phase proteins and an increased baseline effector T cell to regulatory T cell gene expression ratio. Thus, atezolizumab has promising activity in mRCC patients with an excellent safety profile.

### Bladder cancer

In the USA, approximately 76,960 new cases of bladder cancer are detected with an estimated 16,390 deaths in the year 2016 [3]. About half of patients who present with localized tumor can be managed by surgical treatment and these patients have an excellent 5-year survival rate of 96% [3]. Early stage urinary bladder cancer is treated by local therapies including transurethral resection of bladder tumor (TURBT) and intravesical bacillus Calmette–Guérin (BCG) vaccine. Atezolizumab a PD-1 antibody was the first agent approved by the FDA to treat metastatic or advanced bladder cancer after progression on platinum-based combination chemotherapy.

## FDA-approved agents

### Non-muscle-invasive bladder cancer

#### BCG

BCG is a live attenuated vaccine prepared from *Mycobacterium bovis* with immunomodulatory activity. Intravesical BCG is the first-line therapy for non-muscle-invasive (superficial) bladder cancer (T1 and Tis). In the initial randomized study with BCG, 37 patients were randomized to standard surgery or surgery followed by BCG, given once every week for 6 weeks. Eight of 19 control patients (42%) and three of 13 patients (17%) treated with BCG developed recurrent tumors [22]. A number of studies in localized bladder cancer patients showed response rates ranging from 58 to 88% depending on type and stage of tumor, dose of BCG, and median follow-up duration [23–26]. Several studies for prevention of recurrent superficial bladder cancer were performed in which, intravesical BCG was compared with different cytotoxic agents like mitomycin C, thiotepa, doxorubicin, and epirubicin. Intravesical BCG had better outcomes compared to these agents and is thus, the standard of care for non-muscle-invasive bladder cancer after transurethral resection [26–29]. BCG instillation leads to release of multiple cytokines and multiple inflammatory mediators, which attract and activate neutrophils, macrophages, and T cells [30]. These have a potent anti-cancer effect and preserve immunological memory to protect from recurrences. Common side effects associated with BCG are dysuria, hematuria, low-grade fever, and rarely systemic infections.

### Muscle invasive/metastatic bladder cancer

Cisplatin-based combination chemotherapy is the preferred first-line treatment for metastatic urothelial carcinoma. Till recently, there were no FDA-approved second-line therapies and patients usually received single-agent chemotherapies in the USA. Multiple studies have shown that patients with metastatic urothelial carcinoma who have progressed after first-line platinum-based therapy have a median PFS of 2–4 months and median OS of 6–10 months [31]. The results from the IMvigor210 registration study for atezolizumab, which was recently approved for treatment of platinum-resistant bladder cancer are summarized below.

#### Atezolizumab

Atezolizumab (MPDL3280A), an anti-PD-L1 agent, was initially evaluated in a large, phase I study with an expansion cohort for urothelial bladder cancer. Tumors were stratified based on PD-L1 positivity defined as ≥5% of tumor-infiltrating immune cells or tumor cells based on IHC staining. In 67 evaluable patients, the ORR was 43% for PD-L1-positive cohort and 11% for PD-L1-negative cohort. A small proportion of patients 7% in the PD-L1-positive cohort had a complete response with several patients having durable responses. Based on these results, atezolizumab was granted a breakthrough status for bladder cancer by the FDA [32]. Long-term results presented subsequently showed that the median OS in 63 evaluable patients was 28.9 months and median PFS was 5.6 months. Overall, atezolizumab was well tolerated and an increased abseline effector T cell to regulatory T cell ratio was associated with a better response [21].

A multicenter, single-arm, phase II trial evaluated atezolizumab in patients with platinum resistant (IMvigor210). A total of 310 patients with locally advanced or metastatic urothelial carcinoma received atezolizumab 1200 mg IV every 3 weeks [33, 34]. Expression of PD-L1 on tumor-infiltrating immune cells (IC) was measured by immunohistochemistry and classified as IC2/3 with ≥5% staining IC1 with ≥1–4% staining and IC0 with <1% staining. Median OS were 11.4 months (95% CI, 9—not estimable) in IC2/3, 6.7 months in IC1, and 6.5 months in IC0 patients. Median PFS was 2.1 months in all patients, and ORR was 15%. ORR was assessed by PD-L1 IC expression (IC2/3 27% [95% CI] 19–37, *p* < 0.0001; IC1/2/3: 18%, [95% CI] 13–24, *p* = 0.0004). Common side effects with atezolizumab were fatigue, nausea, decrease appetite, and pruritus. Grade 3–4 AEs were uncommon and include fatigue (2%), anemia, and hypertension. Grade 3–4 immune-mediated AEs are pneumonitis, increased aspartate aminotransferase (AST), increased alanine aminotransferase (ALT), rash, and dyspnea being the most common. In this pivotal study, the authors noted that the ORR was much higher for all patients ~15% compared to historical control with ORR of 10%. The authors investigated the role of mutational load as a predictive marker for response and noted that responders had a higher median mutational load of 12 × 4 per megabase compared to non-responders with 6 × 4 per megabase. Unlike lung cancer, smoking was not associated with a higher mutational load and did not predict response to atezolizumab. Using The Cancer Genome Atlas (TCGA) subtyping for bladder cancer based on gene expression profiling, higher response rates were seen in the luminal II subtype. This subtype of bladder cancer is associated with the presence of activated T cells in the tumor. Thus, atezolizumab has excellent activity in platinum-resistant advanced or metastatic urothelial carcinoma and is now approved by the FDA for use in this population. A large phase III study comparing atezolizumab to chemotherapy in bladder cancer after progression on platinum-based chemotherapy is currently ongoing (NCT02302807).

#### Pembrolizumab (MK-3475) for advanced urothelial cancer

This phase Ib trial evaluated pembrolizumab, administered at a dose of 10 mg/kg every 2 weeks in patients with metastatic, recurrent urogenital tract cancers. In this phase Ib study, a total of 33 patients with bladder cancer with PD-L1 expression in stroma or ≥1% tumor cells were enrolled [35]. After 13 month follow-up duration, ORR was 24% (95% CI 11–45), with 3 (10%) complete and 4 (14%) partial responses to pembrolizumab. The 12-month PFS was 19%. Grade 3 or 4 AEs occurred in 15%. The authors concluded that pembrolizumab demonstrates significant antitumor activity in patients with PD-L1-positive bladder cancers. The registration phase III study of pembrolizumab compared to investigator’s choice of chemotherapy (docetaxel, paclitaxel, or vinflunine) in patients with advanced or metastatic bladder cancer has completed accrual, and results are pending (NCT02256436). A number of combination therapies of pembrolizumab with cytotoxic agents (cisplatin, gemcitabine) or targeted therapies for first-line and salvage therapies are currently being evaluated in clinical studies.

#### Durvalumab (MED14736) for advanced urothelial cancer

A phase 1/2, open-label study evaluated durvalumab, an anti-PD-L1 antibody in 61 patients with advanced or metastatic transitional cell carcinoma of the urinary bladder. The overall response rate was 31% in 42 evaluable patients. Median duration of response was not reached yet. Using a unique algorithm, optimized in other malignancies, PD-L1 positivity was defined if ≥25% tumor cells or ≥25% immune cells expressed PD-L1. Interestingly, using this definition of PD-L1 positivity, ORR was much higher 46% in the PD-L1-positive sub-group and 0% in the PD-L1-negative sub-group [36]. Overall treatment with durvalumab was very well tolerated with fatigue, diarrhea, and poor appetite being common treatment-related adverse events.

#### Phase II trial of gemcitabine + cisplatin + ipilimumab (Ipi) in patients with metastatic urothelial cancer

A phase II clinical trial was performed in patients with metastatic urothelial cancer to evaluate the efficacy of ipilimumab (anti-CTLA-4 antibody) in combination with cytotoxic chemotherapy.

A total of 36 patients with metastatic urothelial cancer were enrolled and treated with 2 cycles of gemcitabine and cisplatin (GC) followed by 4 cycles of gemcitabine, cisplatin, and ipilimumab [37]. Primary endpoint of this trial was overall survival at 1 year. The overall response rate (ORR) was 64%, and median OS was around 14.6 months. Median PFS was 8 months (95% confidence interval (CI), 6.2–9.8). Grade 3 or 4 side effects included neutropenia, thrombocytopenia, anemia, hyponatremia, thromboembolism, and renal failure. The immune-related side effects included colitis (6%), hypophysitis (3%), hyperthyroidism (1%), and rash (1%).

#### Pre-operative ipilimumab as window of opportunity study

This elegant pilot trial studied the effects of ipilimumab on bladder cancer in a pre-surgical group of patients [38]. In this trial, 6 patients with localized urothelial carcinoma of the bladder were treated with 3 mg/kg/dose and 6 patients with 10 mg/kg/dose of ipilimumab. Their results showed that ipilimumab use was associated with a higher frequency of CD4 + ICOShi T cells in the tumor and peripheral blood with the 10 mg/kg/dose and this correlated with improved overall survival. Grade 1–2 rash and diarrhea were common side effects. Thus, ipilimumab was noted to have a good safety profile in the pre-surgical setting.

### Prostate cancer

In the USA, approximately 180,890 new cases of prostate cancer were detected with an estimated 26,120 deaths in the year 2016 [3]. Prostate cancer is the most common cancer in men and second most common cause of mortality in men [3]. Due to early detection of prostate cancer by PSA, patients have a 5-year survival rate of around 99% [3]. Localized prostate cancer is treated by surgery (radical prostatectomy) or androgen deprivation therapy (ADT) in combination with external beam radiation therapy (EBRT). Patients with metastatic disease who progress on ADT (castrate-resistant disease) have a poor prognosis and treatment options include oral hormonal agents, chemotherapy, radiotherapy, or immunotherapy.

#### FDA-approved agent: castrate-resistant disease

##### Sipuleucel-T

Sipuleucel-T is a novel cancer vaccine; it contains dendritic or antigen presenting cells (APC), activated using a fusion protein (PA2024) consisting of prostatic acid phosphatase (PAP) and granulocyte-macrophage colony-stimulating factor (GM-CSF) [39]. In the phase I study, 13 patients were treated with two infusions, 1 month apart, of autologous dendritic cells (APC8015) preexposed ex vivo to PA2024, followed by three doses every month of PA2024 subcutaneously. Overall, the treatments were very well tolerated with side effects including grade 1–2 fever, chills, myalgia, local reaction, and fatigue. Antibodies to GM-CSF and PAP were detectable in a number of patients and PSA levels dropped in three patients [39]. A placebo-controlled phase III study was performed with 82 patients with castration-resistant prostate cancer (CRPC) randomized to receive 3 cycles of sipuleucel-T and 45 patients placebo. There was no improvement in the median time to disease progression 11.7 weeks with sipuleucel-T compared with 10.0 weeks for placebo. However, median overall survival, a secondary endpoint improved from 21.4 months with placebo to 25.9 months with sipuleucel-T [40]. The median ratio of T cell stimulation was eightfold higher in sipuleucel-T-treated patients compared to baseline [40]. To confirm these findings that sipuleucel-T improved overall survival, a large double-blind, placebo-controlled, multicenter phase III trial (IMPACT) was designed for men with metastatic CRPC [41]. A total of 512 patients with metastatic CRPC were randomized 2:1 to receive sipuleucel-T (*n* = 341) or placebo (*n* = 171) intravenously every 2 weeks for 3 cycles. The median OS (primary endpoint) was 25.8 months with sipuleucel compared to 21.7 months with placebo and confirmed previous results with a 4.1-month improvement in median OS. The HR for death with sipuleucel-T vs. placebo was 0.78 (95% CI, 0.61–0.98; *p* = 0.03) with a 22% relative reduction in the risk of death. Sipuleucel-T therapy was commonly associated with chills, fever, fatigue, back pain, and headache. Grade 3 or 4 adverse events were uncommon and included chills, back pain, hypokalemia, muscle weakness, and one patient with catheter-related bacteremia. Cerebrovascular events were seen in 8 of 338 patients (2.4%) in the sipuleucel-T group and 3 of 168 patients (1.8%) in the placebo group [41].

Interestingly in both phase III, there was no difference in median time to objective disease progression or time to clinical progression. This may be explained by a delay in the onset of humoral immune responses after immunotherapy and was also consistent with studies with other immunotherapies for CRPC and other cancers [42]. Since immunotherapy vaccines can induce humoral responses to non-targeted tumor antigens, an elegant retrospective study evaluated this antigen spread in 142 patients enrolled on the IMPACT study. They observed elevated IgG levels against multiple secondary antigens, including PSA, after treatment sipuleucel-T, which correlated with sipuleucel-T efficacy. This antigen spread was not observed in patients on the placebo arm and this was specific to sipuleucel-T therapy [43]. In a retrospective analysis of the IMPACT trial, patients with a low-baseline PSA and thus, low overall burden of disease had the best response to sipuleucel-T [44]. Currently, sipuleucel-T is FDA approved for the treatment of patients with metastatic CRPC with no or minimal symptoms. There have been concerns over the use of logistics and cost associated with each use of sipuleucel-T (~$35000 per cycle). As compared to the USA, the National Institute for Health and Care Excellence (NICE) noted that the incremental cost-effectiveness ratio (ICER) for sipuleucel-T was high and not cost-effective and thus did not recommend sipuleucel-T therapy for minimally or asymptomatic patients with metastatic CRPC [45].

#### Other agents

##### PROSTVAC

PROSTVAC is a recombinant vaccinia virus encoding the human PSA. In a phase I study, PROSTVAC was administered to 33 men with prostate cancer at three doses. Ten patients who received the highest dose of PROSTVAC-V also received granulocyte-macrophage colony-stimulating factor (GM-CSF) as an immuno-stimulatory molecule. A majority of patients (82%) developed mild local reaction after the first dose. A single patient developed grade 3 fever and tachycardia with PROSTVAC-V + GM-CSF. Nineteen patients had a PSA reduction at some point during study, and nine patients had PSA stabilization for 11–21 months after study treatment. No IgG to PSA were detectable in these patients; however, specific T cell responses were observed in 5 of 7 patients in the combination arm [46]. A subsequent phase I study evaluated PROSTVAC-V followed by a booster recombinant fowlpox virus (PROSTVAC-F) in combination with co-stimulatory molecules B7-1, ICAM-1, and LFA-3 (designated TRICOM™).

A total 10 patients with castrate resistant prostate cancer with or without metastatic disease were enrolled to evaluate the safety and immunogenicity of this combination [47]. Four patients developed PSA stabilization defined as (less than 25% increase in PSA during the 8-week study period). Anti-vaccinia titers were increased in all patients but as seen in previous and subsequent studies none of the patients developed any anti-PSA antibody response. Common side effects were injection site reactions and fatigue with grade 3 or 4 adverse events [47].

In a phase II, double-blind study, 125 patients with minimally symptomatic metastatic CRPC were randomized in a 2:1 ratio to receive PROSTVAC-VF (*n* = 82, vaccinia-based vector followed by six fox pox-based vector boosts plus granulocyte-macrophage-colony-stimulating factor) or control (*n* = 40, empty vector plus saline injections) [42]. At 3 years, patients treated with PROSTVAC-VF demonstrated a higher OS than control group (30 vs. 17%); median OS was prolonged by 8.5 months (25.1 vs. 16.6 months) and had a substantial reduction in the risk of death by 44%. Common side effects were erythema, pain, and itching at local injection site and fatigue, fever, chills, nausea, and dizziness. The major grade 3 side effects include cellulitis, thrombotic thrombocytopenic purpura, and myocardial infarction. A large randomized, double-blind, phase III study with PROSTVAC-VF was just completed in men with asymptomatic or minimally symptomatic metastatic CRPC (PROSPECT study). Nearly 1298 men were randomized 1:1:1 to PROSTVAC-VF-TRICOM with GM-CSF (arm 1), PROSTVAC-VF-TRICOM with placebo (arm 2) or placebo alone (arm 3) (NCT01322490). This study was powered to evaluate overall survival as their primary endpoint and results are awaited.

##### Ipilimumab

Ipilimumab, anti-CTLA-4, was evaluated in randomized, multicenter, double-blind, phase III study, in patients with at least one bone metastasis from castration-resistant prostate cancer (mCRPC) previously treated with docetaxel. In this study, all 799 patients had received bone-directed radiotherapy (8 Gy in one fraction) and randomized in 1:1 ratio to receive ipilimumab 10 mg/kg of body weight (*n* = 399) vs. placebo (*n* = 400) every 3 weeks up to 4 doses [48]. The median OS (primary endpoint) was 11.2 months with ipilimumab compared to 10.0 months with placebo (HR 0.85, 0.72–1.00; *p* = 0.053). Ipilimumab was commonly associated with diarrhea, pruritus, and rash. Grade 3–4 adverse events commonly seen with ipilimumab include diarrhea, fatigue, anemia, and colitis. Four deaths (1%) in the ipilimumab arm were attributed to the study treatment; however, an increased number of patients 73 (19%) in the ipilimumab arm died in the initial 5 months compared to the placebo arm with 53 deaths (13%). Ipilimumab use was associated with reduction in PSA (13.1% patients) and improvement in progression free survival but failed to improve their overall survival, the primary endpoint of this study. In a post-hoc analysis, favorable prognostic features (defined as having no visceral disease, no anemia, and normal to mildly elevated alkaline phosphatase) were associated with improved overall survival [48].

##### PROSTVAC in combination with ipilimumab

Ipilimumab and PROSTVAC vaccines were given to mCRPC patients in phase I study. In this phase I study, a total of 30 patients with mCRPC were enrolled [49]. Grade 3 or 4 immune-related AEs were diarrhea, rash, raised aminotransferases, endocrine immune-related adverse events, and neutropenia. The use of a PROSTVAC enhances co-stimulation of the immune system but did not exacerbate the immune-related AEs associated with ipilimumab.

##### Checkpoint inhibitors in prostate cancer

In a phase 1, dose-escalation study, 296 patients with advanced melanoma, lung cancer, kidney cancer, colorectal cancer, or castrate-resistant prostate cancer (CRPC)-received nivolumab. No objective responses were seen in 17 patients with metastatic CRPC [18]. As outlined above, subsequent studies with ipilimumab in prostate cancer were also negative; thus, checkpoint inhibitors were not investigated in larger phase 2 or 3 studies in prostate cancer. A recent single-arm, phase II study evaluated the use of pembrolizumab 200 mg intravenously in patients with CRPC, who had progressed on enzalutamide (an androgen pathway inhibitor). The investigators noted a rapid PSA response in three of 10 subjects enrolled so far with two subjects with measurable disease having a partial response. Based on these results, role of checkpoint inhibitors in treatment of prostate cancer needs to be re-evaluated [50].

##### Vaccines in prostate cancer

A number of vaccine strategies are under development for treatment of prostate cancer; these include dendritic cell vaccine (e.g., sipuleucel-T), vector-based vaccine (e.g., PROSTVAC), or whole tumor cell vaccines (e.g., GVAX platform from Cell Genesys, Inc.). An allogeneic cancer vaccine using the GVAX platform with two prostate cancer cell lines PC-3 and LNCaP, genetically modified to secrete granulocyte-macrophage colony-stimulating factor (GM-CSF) was evaluated in 55 patients with biochemical recurrence (21 subjects) or castrate-resistant disease (34 subjects). Median overall survival was higher in both sub-groups compared to historical controls, and the treatments were well tolerated [51].

INO-5150 is a novel plasmid-based DNA vaccine that encodes prostate-specific antigen (PSA) and prostate-specific membrane antigen (PSMA). A recent phase I trial study combined INO-5150) with a plasmid encoded adjuvant IL-12 (INO-9012). The combination was well tolerated with four patients experiencing grade 3 serious adverse events which included hospitalization for fall, transaminitis, pre-syncope, and cardiac disorder [52]. No Grade 4–5 adverse events were noted and efficacy analyses are pending.

### Testicular cancer

In the USA, approximately 8700 new cases of testicular cancer are detected with an estimated 380 deaths in the year 2016 [3]. In recent years, incidence rate of testicular cancer is increasing [3]. Testicular cancer patients have a 5-year survival rate of around 97% [3]. Testicular germ cell tumors (TGCTs) are highly chemo and radiosensitive malignancies accounting for the high-cure rates. Currently, there are no FDA-approved agents for the treatment of testicular malignancies.

In a retrospective study, immunohistochemistry was performed on testicular germ cell tumors (TGCT) to evaluate for programmed death receptor ligand-1 (PD-L1) expression. Using a rabbit monoclonal antibody, PD-L1 expression was seen in 73% of all seminomas and in 64% of all non-seminomas but none of the normal testicular tissue [53].

A second study conformed that PD-L1 expression was higher in TGCTs compared to normal testicular tissue (QS = 5.29 vs. 0.32, *p* < 0.0001) [54]. Choriocarcinoma expressed the highest level of PD-L1 followed by embryonal carcinoma, teratoma, yolk sac tumor and seminoma. Patients, who had tumors with low-PD-L1 expression had a better PFS with a hazard ratio ((HR) = 0.40, *p* = 0.008). Overall survival in patients with low-PD-L1 expression was also improved with a hazard ratio ((HR = 0.43, *p* = 0.04) compared to patients with high-PD-L1 expression. There is a well-designed, ongoing phase II clinical study studying the role of pembrolizumab in patients with recurrent or metastatic germ cell tumor, which is cisplatin resistant (NCT02499952) (Table 1).

**Table 1:** Completed phase II or III clinical studies in genitourinary malignancies

Study	Indication	Dose	Mechanism of action	Result	Common AE
HD-IL2 [11]	Metastatic RCC, first line	600,000 or 720,000 IU/kg every 8 hourly up to 14 consecutive doses for 5 days every 2 weeks. Four to 6 cycles of treatment based on clinical and radiographic responses	HD-IL2 stimulates proliferation and differentiation of T, B, and NK lymphocytes. It causes recruitment of tumor-infiltrating lymphocytes at tumor sites	ORR was 14% with complete response (CR) seen in 5% patients and partial response (PR) in 9% of patients. Median duration of PR was 19 months	Capillary leak syndrome, hypotension, fever and chills, anemia, nausea and vomiting, diarrhea, mental status changes, elevated liver enzymes and bilirubin, elevated BUN and creatinine, dyspnea, and pruritus
IFN-α plus bevacizumab vs. IFN-α [14, 15]	Metastatic RCC, first line	IFN-α (9 million units SC 3 times/week) with bevacizumab (10 mg/kg intravenously every 2 weeks) vs. IFN-α	IFN-α is a cytokine with immune-modulatory and anti-proliferative activity	Median PFS was 8.5 months for bevacizumab plus IFN-α (95% CI, 7.5 to 9.7 months) compared to 5.2 months (95% CI, 3.1 to 5.6 months) for IFN-α monotherapy. Median OS (primary end point) 18.3 months for bevacizumab plus IFN-α compared to 17.4 months for IFN-α monotherapy	Fatigue, anorexia, nausea, proteinuria, neutropenia, and hypertension
Bevacizumab plus IFN-α vs. IFN-α plus placebo [16]	Metastatic RCC, first line	Bevacizumab (10 mg/kg every 2 weeks) with IFN-α (9 MIU SC 3 times/week) or same dose IFN-α with placebo	IFN-α is a cytokine with immune-modulatory and anti-proliferative activity	Median OS (primary end point) with bevacizumab/IFN-α was 23.3 months vs. 21.3 months. At interim analysis, median PFS was significantly longer with bevacizumab/IFN-α vs. IFN-α/placebo (10.2 vs. 5.4 months and ORR 31 vs. 13%, respectively	Fatigue, asthenia, and neutropenia with IFN-α and proteinuria, hypertension, GI perforation, and bleeding with bevacizumab. There were 2% deaths related to treatment on both arms.
Nivolumab vs. everolimus [18]	Metastatic RCC, Second line and beyond	Nivolumab 3 mg/kg of body weight every 2 weekly vs. oral 10 mg everolimus tablet daily.	Programmed death 1 (PD-1) checkpoint inhibitor	Median OS was 25.0 months with nivolumab compared to 19.6 months with everolimus. Median PFS 4.6 months with nivolumab compared to 4.4 with everolimus. The ORR was superior with nivolumab than everolimus	Nivolumab arm: fatigue, nausea, pruritus, diarrhea, anorexia, and rash
Atezolizumab [33, 34]	Metastatic or advanced bladder cancer after platinum-based chemotherapy	1200 mg fixed dose intravenous every 3 weeks	Programmed death-1 ligand (PD-L1) inhibitor	ORR 15% in all patients with a complete response rate of 5%. Median duration of response not reached. Median PFS was 2.1 months and median OS 7.9 months	Fatigue, nausea, poor appetite pruritus, anemia, hypertension, pneumonitis, increased AST, ALT, rash, and dyspnea
Sipuleucel-T vs. placebo [40, 41]	Metastatic CRPC	Three cycles intravenously every 2 weeks	Dendritic cells activated using a fusion protein (PA2024) consisting of prostatic acid phosphatase (PAP) and GM-CSF	Median OS was 25.8 months with sipuleucel compared to 21.7 months with placebo. PFS was not different between the two arms	Sipuleucel-T: chills, fever, fatigue, back pain, and headache

### Penile cancer

In the USA, approximately 2000 new cases of penile cancer are detected with an estimated 340 deaths in the year 2016 [3]. In a retrospective study, 23 samples (penile cancer and/or lymph nodes) were collected from 19 patients with squamous cell carcinoma of the penis. PD-L1 expression was evaluated by IHC using a H-score of >5% as positive and 5 of 23 samples (22%) tested positive for PD-L1 expression [55]. In a separate study, Twenty-three (62.2%) of 37 primary penile squamous cell carcinoma tumors tested positive for PD-L1 expression. PD-L1 expression was associated with advanced disease, nodal metastases, and reduced disease specific survival [56]. Multiple studies evaluating checkpoint inhibitors for advanced penile cancer are currently ongoing (Table 2).

**Table 2:** Selected ongoing clinical studies in patients with genitourinary malignancies

No.	Study	Disease type	Intervention/dose	Mechanism of action	Study phase and sponsor	Primary endpoints
1.	A Phase I Study of Hyperacute®-Renal (HAR) Immunotherapy In Patients With Metastatic Renal Cell Cancer (NCT02035358)	Metastatic renal cell carcinoma	Cells injected intradermally every week × 4 weeks and then every 2 weeks for 10 immunizations to total 14 immunizations. Dose cohort 1:150 million cells per immunization; Dose cohort 2: 300 million cells per immunization.	Two allogeneic renal cancer cell lines expressing murine α1,3 galactosidase gene	Phase 1, NewLink Genetics	Toxicity, DLT, and MTD
2.	Neoadjuvant AGS-003 Immunotherapy in Patients With Localized Kidney Cancer (NCT02170389)	Newly diagnosed advanced renal cell carcinoma, prior to nephrectomy or metatasectomy	AGS-003 with sunitinib	CD40L RNA-transfected autologous dendritic cell vaccine	Phase 2, Argos Therapeutics	Changes in immune biomarkers
3.	Adjuvant Antigen Specific Immunotherapy in Patients With Advanced Renal Cell Carcinoma Using Tumor Associated Peptides (NCT02429440) UroRCC	Renal cell carcinoma after resection or metatasectomy	Arm 1: Intradermal application of peptide vaccine in combination with granulocyte macrophage colony-stimulating factor (GM-CSF) Arm 2: Intradermal application of peptide vaccine with Montanide ISA-51	Synthetic adjuvant peptide with immune boosters	Phase 1 and 2, University Hospital Tuebingen	Safety and tolerability
4.	Phase I Study of Neoadjuvant Nivolumab in Patients With Non-metastatic High-risk Clear Cell Renal Cell Carcinoma (NCT02575222)	Clear cell renal cell carcinoma prior to nephrectomy	Nivolumab at 3 mg/kg, IV (in the vein) on day 1 of each 2-week cycle, for a total of 3 doses prior to nephrectomy	PD-1 inhibitor	Phase 1, Sidney Kimmel Comprehensive Cancer Center	Safety and tolerability
5.	A Phase I/Ib, Open Label, Dose Finding Study to Evaluate Safety, Pharmacodynamics and Efficacy of Pembrolizumab in Combination With Vorinostat in Patients With Advanced Renal or Urothelial Cell Carcinoma (NCT02619253)	Renal cell carcinoma or urinary bladder cancer	Pembrolizumab and vorinostat	Pembrolizumab: anti-PD-1 antibody and vorinostat is a histone deacetylase inhibitor	Phase 1 and 2, Indiana University	Maximum tolerated dose (MTD) or recommended phase 2 dose (RP2D)
6.	Phase Ib Trial Of Pembrolizumab And Nintedanib (NCT02856425)	Patients with any advanced solid tumors.	Nintedanib pembrolizumab	Pembrolizumab: anti-PD-1 antibody and nintedanib is a tyrosine kinase inhibitor to VEGF, FGFR, and PDGFR	Phase 1, Gustave Roussy, Cancer Campus, Grand Paris	Safety and MTD of the combination
7.	A Phase Ib/II Study of ALT-801 in Patients With Bacillus Calmette-Guerin (BCG) Failure Non-muscle Invasive Bladder Cancer (NCT01625260)	Non-muscle invasive bladder cancer	ALT-801 gemcitabine	ALT-801 is a recombinant protein, where IL2 is fused to T cell receptor directed to p53	Phase 1 and 2, Altor Bioscience Corporation	Safety/efficacy study
8.	The Effect of Atezolizumab in Combination With Gemcitabine/Carboplatin and Gemcitabine/Carboplatin Alone in Participants With Untreated Locally Advanced or Metastatic Urothelial Carcinoma Who Are Ineligible for Cisplatin-based Therapy [IMvigor130] (NCT02807636)	Locally advanced or metastatic urothelial cancer, cisplatin ineligible patients for 1^st^ line therapy	Arm A: atezolizumab 1200 mg every 3 weeks with carboplatin AUC 4.5 day 1 every 3 weeks and gemcitabine 1000 mg/m2 days 1 and 8 every 3 weeks Arm B: carboplatin with gemcitabine	Atezolizumab is a programmed death-1 ligand (PD-L1) inhibitor	Phase 3	Efficacy, PFS, and OS
9.	Randomized Phase 2 Trial of ACP-196 and Pembrolizumab Immunotherapy Dual CHECKpoint Inhibition In Platinum Resistant Metastatic Urothelial Carcinoma (RAPID CHECK Study) (NCT02351739)	Metastatic Urothelial Carcinoma	Arm 1: pembrolizumab Arm 2: ACP-196 in combination with pembrolizumab	Pembrolizumab is a PD-1 inhibitor Acalabrutinib (ACP-196) is as irreversible inhibitor of Bruton’s tyrosine kinase(BTK)	Phase 2 Acerta Pharma	Efficacy and safety
10.	Phase I, Open-label Trial to Evaluate the Safety and Immunogenicity of INO-5150 Alone or in Combination With INO-9012 in Men With Biochemically Relapsed Prostate Cancer (NCT02514213)	Biochemical or PSA recurrence of prostate adenocarcinoma	Arm1: 2 mg INO-5150 Arm2: 8.5 mg INO-5150 Arm3: 2 mg INO-5150 plus 1 mg INO-9012 Arm4: 8.5 mg INO-5150 plus 1 mg INO-9012 Intramuscular delivery using electroporation	INO-5150 is a plasmid DNA vaccine encoding PSA and prostate-specific membrane antigen (PSMA). INO-9012 is an IL2 immune activator	Phase 1 Inovio Pharmaceuticals	Safety
11.	A Randomized, Placebo-Controlled Phase II Study of Multi-Epitope TARP Peptide Autologous Dendritic Cell Vaccination in Men With Stage D0 Prostate Cancer (NCT02362451)	Biochemical or PSA recurrence of prostate adenocarcinoma	Arm 1:lead in cohort ME TARP vaccine Arm 2: experimental arm ME TARP vaccine Arm 3:Autologus monocyte placebo	ME TARP is a multi-epitope T cell-receptor alternating reading frame protein expressed in 90-95% prostate cancer cells	Phase II National Cancer Institute	Safety and efficacy
12.	Biomarker-Driven Therapy With Nivolumab and Ipilimumab in Treating Patients With Metastatic Hormone-Resistant Prostate Cancer Expressing AR-V7 (STARVE-PC) (NCT02601014)	Metastatic CRPC patients with detectable AR‐V7 transcript in circulating tumor cells	Nivolumab in combination with ipilimumab	Nivolumab is a PD-1 inhibitor and ipilimumab is an anti-CTLA-4 antibody	Phase 2, Johns Hopkins University/Sidney Kimmel Cancer Center	Efficacy and safety
13.	Docetaxel and PROSTVAC for Metastatic Castration Sensitive Prostate Cancer (NCT02649855)	Metastatic CRPC	Arm A: standard ADT followed by simultaneous docetaxel and PROSTVAC Arm B: standard ADT followed by sequential docetaxel and PROSTVAC	PROSTVAC is a recombinant vaccinia virus encoding the human PSA	Phase 2 National Cancer Institute	Biomarker, evaluating antigenic spreading and response at 19 weeks
14.	A Phase II Single-Arm Multi-Center Trial Evaluating the Efficacy of Pembrolizumab in the Treatment of Subjects With Incurable Platinum-Refractory Germ Cell Tumors (NCT02499952)	Incurable platinum-refractory germ cell tumors	Pembrolizumab 200 mg every 3 weeks	Anti PD-1 inhibitor	Phase 2 Hoosier Cancer Research Network GU14-206	Safety and efficacy study
15.	A Phase II Clinical Trial of Single Agent Pembrolizumab in Subjects With Advanced Adrenocortical Carcinoma (NCT02673333)	Unresectable or metastatic adrenocortical carcinoma	Pembrolizumab 200 mg every 3 weeks	Anti-PD-1 inhibitor	Phase 2 Memorial Sloan Kettering Cancer Center	Safety and efficacy study

Derived from Clinical trials; A service of the U.S. National Institutes of Health; Retrieved from https://clinicaltrials.gov/ct2/home

### Adrenocortical carcinoma

Adrenocortical carcinoma is an extremely rare tumor, with advanced disease associated with an extremely poor outcome. The 5-year survival rate in localized, regional, and distal adrenocortical carcinoma are approximately 65, 44, and 7%, respectively [57]. PD-L1 expression was studied using IHC in tumor cell membrane and tumor-infiltrating mononuclear cells (TIMC) for 28 patients with adreno-cortical carcinoma. Three of 28 patients (10.7%) were positive for PD-L1 expression in tumor cell membrane and 19 of 27 (70.4%) for tumor-infiltrating mononuclear cells. However, PD-L1 positivity did not correlate with higher stage, grade, or overall survival [58]. Biological agents and targeted therapy are under clinical trials (Table 2).

## Biomarkers of response

1. PD-L1 expression in tumor cells and tumor-infiltrating cells: There is some debate on the prognostic and predictive role of PD-L1 immunohistochemistry in GU malignancies. A meta-analysis evaluated 1475 cancer patients treated with PD-1 or PD-L1 inhibitors and noted a clinical response in 34.1% patients with PD-L1-positive tumors and 19.9% PD-L1-negative tumors. For GU malignancies (renal and bladder), the difference in response rates between PD-L1-positive or PD-L1-negative malignancies was not statistically significant [59]. There are a number of issues, which remain unaddressed to validate PD-L1 positivity as a predictive marker. Collecting achieved tissue provides us with a snapshot of PD-L1 status; however, this status is dynamic and may change depending on site, time of biopsy, and concomitant anti-tumor agents. Also, there is great variability on PD-L1 positivity based on the type of antibodies used for staining and the cut-off used to define PD-L1 positivity.
2. Mutational load: In an elegant study, Alexandrov and colleagues studied the number of mutations and mutational signatures in a variety of cancers [60]. Tumors with a high mutational load like bladder cancer, melanoma, and lung cancer demonstrate a very high response rate to checkpoint inhibitors [60]. Interestingly, a number of patients with renal cell carcinomas have an excellent response to checkpoint inhibitors in spite of having a low mutational burden.
3. Neoantigens: Tumor-specific mutant antigens or neoantigens are specific protein epitopes present on tumor cells, which form an important target for checkpoint inhibitors [61]. With recent innovation in molecular biology and genetics, it is possible to identify the immune response to neoantigens that derived from tumor-specific mutations. In a study with melanoma patients treated with ipilimumab, exomes and transcriptome data was obtained from a pretreatment melanoma tissue sample (*n* = 110). The investigators noted that mutational load, neoantigens, and expression of cytolytic markers were predictive markers associated with clinical benefit to ipilimumab [62, 63].
4. Activation of the WNT/β-catenin pathway by either mutations or increased expression occurs in a number of malignancies. This correlates with T cell exclusion and may predict poor response to immunotherapy [64].

## Conclusions

Immunotherapies have expanded the treatment options available for patients with genitourinary malignancies. With the availability of checkpoint inhibitors, durable responses are seen in patients with metastatic platinum-resistant urothelial carcinomas, who had limited options before. Nivolumab use in metastatic renal cell carcinoma is associated with a significant improvement on overall survival and meaningful improvement in the quality of life. A number of vaccines and checkpoint inhibitor combination trials are currently ongoing and are highlighted in Table 2. The dosing for checkpoint inhibitors was based on body size, and these agents are packaged in single-dose vials. This leads to substantial amount of drug wastage and unnecessary overspending [65]. A number of these agents (pembrolizumab, atezolizumab, and nivolumab) are now being evaluated with fixed dosing aimed to reduce drug waste. There are a number of questions, which need to be looked into; these include development of predictive biomarkers, the duration of therapy with checkpoint inhibitors, and whether there may be a rationale for maintenance therapy with these agents. Thus, the field of immunotherapy for genitourinary malignancies in constantly evolving and has significantly impacted the treatment of these malignancies.

## Abbreviations

ADT: Androgen deprivation therapy
APC: Antigen presenting cells
BCG: Bacillus Calmette–Guérin
CA-9: Carbonic anhydrase-9
CR: Complete response
CTLA-4: Cytotoxic T-lymphocyte associated antigen 4
EBRT: External beam radiation therapy
ECOG: Eastern Cooperative Oncology Group
FDA: Food and Drug Administration
GM-CSF: Granulocyte-macrophage colony-stimulating factor
HD-IL2: High-dose interleukin
HR: Hazard ratio
IFN-α: Interferon alpha
IHC: Immunohistochemistry
LFA-3: Lymphocyte function-associated antigen 3
mCRPC: Metastatic castration-resistant prostate cancer
mRCC: Metastatic renal cell carcinoma
ORR: Overall response rate
PA2024: Activated using a fusion protein
PAP: Prostatic acid phosphatase
PD-1: Programmed death 1
PD-L1: Programmed death ligand 1
PFS: Progression free survival
PR: Partial response
PS: Performance status
TCGA: The Cancer Genome Atlas
TGCTs: Testicular germ cell tumors
TIMC: Tumor infiltrating mononuclear cells
TURBT: Transurethral resection of bladder tumor
VEGF: Vascular endothelial growth factor
